# A randomized controlled trial of staged cardiac rehabilitation based on the Health Belief Model on kinesiophobia, self-efficacy and activities of daily living in elderly patients with coronary heart disease

**DOI:** 10.3389/fmed.2026.1857532

**Published:** 2026-06-22

**Authors:** Xiaoqin Zheng, Ya Tan, Xiao Wang, Qi Li, Jinyan Li, Xin Lan, Song Zhang

**Affiliations:** 1 Department of Nursing, Suining Central Hospital, Suining, China; 2 School of Nursing, Zunyi Medical University, Zunyi, China

**Keywords:** aged, cardiac rehabilitation, Health Belief Model, kinesiophobia, self-efficacy

## Abstract

**Introduction:**

Kinesiophobia is highly prevalent in elderly patients with coronary heart disease (CHD) and severely limits participation in cardiac rehabilitation. However, phased, theory-based interventions targeting this population are lacking.

**Methods:**

This single-blind randomized controlled trial of 139 elderly CHD patients evaluated a staged cardiac rehabilitation intervention based on the Health Belief Model (HBM). The intervention group (*n* = 69) received a staged HBM-based programme plus usual care; the control group (*n* = 70) received usual care only. Outcomes (TSK-SV Heart, Barthel Index, SEE) were measured at admission (T0), discharge (T1), and 1 month (T2) and 3 months (T3) post-discharge.

**Results:**

Repeated-measures ANOVA revealed significant time, group, and interaction effects for all outcomes (*P* < 0.05). The intervention group showed significantly higher Barthel Index and SEE scores at T1, T2, and T3, and lower TSK-SV Heart scores at T2 and T3 versus controls (*P* < 0.05). Within the intervention group, kinesiophobia at T2/T3 was lower than at T0/T1, and self-efficacy at T1–T3 was higher than at T0 (*P* < α'). No exercise-related adverse events occurred.

**Discussion:**

A staged HBM-based cardiac rehabilitation programme reduces kinesiophobia, improves ADL, and enhances exercise self-efficacy in elderly CHD patients, with sustained effects and excellent safety. This nurse-led, low-cost programme can be integrated into standard cardiac rehabilitation pathways for older adults.

## 1 Introduction

Coronary atherosclerotic heart disease (CHD) is a major cause of disability and mortality worldwide. In China, the number of CHD patients has exceeded 10 million, with prevalence and disease burden showing a continuous upward trend. The elderly population is a group with a high incidence of CHD and a relatively poor prognosis (1). This is due to declining physiological function and multiple comorbidities. Exercise-based cardiac rehabilitation is a Class IA recommended intervention for the secondary prevention of CHD. It can effectively delay disease progression and reduce the risk of all-cause mortality and readmission (2, 3). However, participation rates and adherence to cardiac rehabilitation remain generally low worldwide, with this issue being particularly pronounced among elderly patients (4). In addition to objective factors such as limited access to healthcare resources and insufficient family support, psychological barriers are a key factor hindering patient participation in rehabilitation, with kinesiophobia being particularly significant (5). This condition stems from the “fear-avoidance” model, referring to patients' excessive and irrational fear of physical activity, characterized by concerns that exercise may trigger adverse cardiac events such as angina or arrhythmias, thereby creating a vicious cycle of “fear–avoidance–functional decline–intensified fear” (6). Meta-analyses indicate that the prevalence of kinesiophobia among CHD patients in China is as high as 61.1%. Elderly patients are more sensitive to perceived disease risks. They exhibit higher levels of kinesiophobia than middle-aged and younger patients (7). This not only reduces their willingness to participate in rehabilitation and adhere to behavioral advice, but also correlates with physical functional decline, reduced ADL, and poor prognosis (8). Therefore, exploring cardiac rehabilitation intervention strategies tailored to the characteristics of elderly CHD patients that can effectively alleviate exercise-related fear holds significant clinical importance.

The Health Belief Model (HBM), as a classic theoretical framework for systematically explaining the formation of health behaviors, comprehensively reveals the psychological pathways of individual health decision-making through six dimensions: perceived susceptibility, severity, benefits, barriers, action cues, and self-efficacy (9). Its core value lies in promoting the formation and maintenance of health behaviors by reshaping individual health beliefs. Currently, this model has been proven to effectively enhance patients' self-management capabilities and health behavior levels in the management of chronic conditions in older adults, such as hypertension and diabetes (10, 11), yet systematic intervention studies based on the HBM targeting exercise-related anxiety in CHD patients remain relatively limited in China; Existing relevant studies are largely confined to evaluating effects at a single time point, and intervention programmes lack a phased design, making it difficult to reveal the dynamic trends in intervention outcomes and failing to meet the gradual rehabilitation needs of elderly patients. Consequently, this study combines the physiological and psychological characteristics of elderly CHD patients to develop a phased, structured cardiac rehabilitation intervention based on the HBM. Employing a repeated-measure design, we dynamically analyze the characteristics and sustained effects of this intervention on patients' exercise anxiety, ADL, and exercise self-efficacy, with the aim of providing comprehensive and objective theoretical and practical evidence for the development of psychologically oriented rehabilitation strategies for elderly CHD patients in clinical practice.

## 2 Subjects and methods

### 2.1 Study population

A randomized controlled trial design was employed, recruiting elderly CHD patients hospitalized at our institution between November 2024 and October 2025 as study subjects. Inclusion criteria: Compliance with the relevant diagnostic criteria of the “Guidelines for the Diagnosis and Treatment of CHD” (12); Age ≥ 65 years; New York Heart Association (NYHA) functional class ≤ Class III; A score >37 on the Tampa Scale for Kinesiophobia Heart (TSK-SV Heart) (13); Alert and oriented, with no psychiatric disorders and normal communication and comprehension abilities; Informed consent and voluntary participation in this study. Exclusion criteria: Patients with severe hepatic or renal insufficiency, hematological disorders, autoimmune diseases, or other serious complications; Individuals currently taking anti-anxiety or anti-depressant medication. Acute coronary syndrome or acute decompensated heart failure within the previous 3 months; Coronary artery bypass grafting within the previous 6 months; Any unstable cardiac condition (e.g., refractory arrhythmias, electrical storm). Censoring criteria: Participants who voluntarily withdrew, were lost to follow-up, or had incomplete data for any reason during the study period. Functional status at baseline: All patients were able to walk independently with or without a walking aid (e.g., cane); none were wheelchair-bound. Approximately 15% of patients used a cane for outdoor mobility, but no patient required a walker or crutches. The TSK-SV Heart score was used as the primary outcome measure. The sample size was calculated using the formula N = [(Z_1−α/2_+ Z_1−β_)^2^* ($\sigma_{1}^{2}+$ $\sigma_{2}^{2}$)] / δ^2^= 2[(Z_1−α/2_+ Z_1−β_(2)^2^ ]/d^2^ and the results of the pilot study, α was set at 0.05, β at 0.1, σ at 3, and δ at 2. Consulting the table, Z_α_ = 1.96 and Z_β_ = 1.28. Taking into account a 10% attrition rate, the sample size for each group was determined to be 65. Participants were randomized into a control group and an intervention group by the director of the nursing department using a randomization tool in the Medsci medical software, with allocation based on odd and even numbers. Allocation concealment was implemented using sequentially numbered, sealed, opaque envelopes to avoid selection bias. A single-blind design was implemented: data collection and scale scoring were conducted by a dedicated data manager not involved in the intervention delivery. Neither the interventionists nor the participants were blinded. This study has fully considered the potential bias arising from the non-blinded design and has mitigated the impact of bias through standardized intervention procedures and strict data collection protocols. This study has been approved by the hospital's ethics committee (KYLLKS20240193) and has completed registration and review with the China Clinical Trials Registry (ChiCTR2500097008).

### 2.2 Intervention methods

#### 2.2.1 Intervention methods for the control group

Routine care for CHD was provided, comprising the following: Admission health education: delivered via verbal instruction and information leaflets, covering the etiology of CHD, medication, diet and basic exercise knowledge; Condition monitoring: routine monitoring of heart rate, blood pressure, oxygen saturation and ECG changes; Medication management: guiding patients to take medication regularly as prescribed, and explaining adverse drug reactions and precautions; Discharge guidance: The attending physician develops a standard exercise plan (e.g. 10–20 min of brisk walking daily) based on the patient's cardiac function classification, provides verbal instruction, and informs the patient of follow-up appointment dates and precautions.

#### 2.2.2 Intervention methods for the experimental group

Establish and train a HBM-based cardiac rehabilitation intervention team. Team members include one specialist doctor, one rehabilitation therapist, one head nurse, two charge nurses and one postgraduate nursing student. All team members hold a bachelor's degree or higher, possess good communication skills with patients and demonstrate a strong sense of responsibility toward research. Upon enrolment, all members received training in health belief theory and specialized knowledge and skills related to exercise-related anxiety. The postgraduate nursing student provided instruction on the principles of scale use and scoring criteria. Only after all team members had passed the training assessment were they permitted to commence work with patients. An HBM-based cardiac rehabilitation protocol was implemented. This protocol was developed following a literature review by the researchers, expert panel deliberation, and a pilot study. It was executed in four phases; specific measures are detailed in Table 1. Detailed exercise prescription (Stage 3): Exercise intensity was guided by the Borg Rating of Perceived Exertion (RPE) scale (6–20), with a target of 11–13 (“fairly light to somewhat hard”). For patients in NYHA class I: walking at 40–50 meters per minute, RPE 11–12, duration 15 minutes per session, twice daily, supplemented by ankle pumping and seated leg exercises. For NYHA class II–III: slow walking at 30–40 meters per minute, RPE 11, duration 10 minutes per session, twice daily, plus bedside sitting-standing exercises. The incremental goal “walk an extra 5 min each day” was applied until the maximum tolerated duration was reached (25 min for class I, 15 min for class II–III by the end of hospitalization).

**Table 1:** HBM-based cardiac rehabilitation intervention protocol.

Time	Item	Implementation
Phase 1: Day1of admission	Assessing vulnerability and severity	**1. One-to-one assessment and trust-building**: The responsible nurse proactively introduces the environment and, through semi-structured interviews with the patient and their family, understands their knowledge of the condition, concerns and previous activity levels, thereby establishing a good nurse-patient relationship. **2. Guiding Risk Perception**: Using plain language and drawing on the patient's medical history, explain the risks associated with the onset and progression of CHD and exercise-induced anxiety, as well as the serious threat they pose to health and quality of life, enabling the patient to recognize the vulnerability and severity of the condition. **3. Baseline Assessment and Joint Goal Setting**: Collect general information and guide the patient in completing the initial TSK-SV Heart, Barthel Index and SEE scales (T0) to determine their level of exercise-related anxiety, functional status and self-efficacy, whilst also exploring their rehabilitation expectations.
Stage 2: Days 2–3 after admission	Overcoming perceptual barriers and enhancing perceived benefits	**1. Group Information Session**: Organize a 60-min specialized lecture for patients and their families, using a PowerPoint presentation to showcase successful clinical cases of cardiac rehabilitation in elderly CHD patients alongside evidence-based research data. This session systematically explains the clear benefits of scientifically guided exercise in improving cardiac function and enhancing Activities of Daily Living (ADL), whilst directly refuting misconceptions such as “exercise will worsen the condition”; **2. Supervised interactive experiential exercise:** Following the lecture, rehabilitation therapists and nurses lead 20-min sessions of low-intensity, safe exercise, including seated breathing exercises (8–10 breaths per minute for 10 min), bedside ankle pumping exercises (10 repetitions of dorsiflexion and 10 of plantar flexion per set, for a total of 2 sets). Vital signs are monitored before, during and after the exercise, and personalized positive feedback is provided promptly afterwards, allowing patients to experience first-hand the feasibility and safety of exercise.
Stage 3: From the 4th day of admission to the day of discharge	Enhancing self-efficacy	**1. Individualized exercise prescription**: Rehabilitation therapists develop an in-hospital individualized exercise plan based on the patient's NYHA cardiac function classification. Class I cardiac function: 15-min walks in the corridor per session, twice daily, at a speed of 40–50 meters per minute; For NYHA Class II–III: bedside sitting and standing exercises + 10-min slow walks, twice daily, at a pace of 30–40 meters per minute, gradually achieving the quantifiable short-term goal of “walking 5 min more each day.” **2. Accumulation of successful experiences and feedback**: Nurses should keep detailed records of patients' exercise completion. After each session, provide specific feedback using encouraging language (e.g., “You completed a 10-min walk under supervision today; your heart rate and blood pressure remained stable, indicating that you can safely perform this activity!”) to reinforce their sense of control and experience of success. **3. Consolidation and Assessment** Prior **to** Discharge: 1 to 2 days before discharge, the patient completes one or two simulated home-environment exercise sessions accompanied by a nurse or family member. This alleviates concerns regarding exercise safety after discharge, boosts confidence in exercising, and provides further guidance on completing the TSK-SV Heart, Barthel Index and SEE scales (T1) to assess short-term changes, whilst offering guidance to reinforce confidence in post-discharge exercise. Safety monitoring is integral to this phase: heart rate, blood pressure, oxygen saturation and subjective symptoms are monitored before, during and after all exercise sessions, and clear “Criteria for Terminating Exercise” are established (e.g., onset of chest pain, dizziness, abnormal rise in blood pressure, etc.).
Phase 4: Within 3 months of discharge	Follow-up and Consolidation Action Plan	**1. Structured, multi-channel follow-up**: **WeChat support group** (for those able to use a smartphone): Group members send out standardized educational content weekly (Week 1: Recognizing signs of safe exercise; Week 2: Home rehabilitation exercise videos (15 min per set); Week 3: Techniques for overcoming inertia; Week 4: Nutritional advice), answer queries in real time, encourage interaction among patients, and provide ongoing action prompts and social support; **Telephone follow-up** (for all patients): Using a semi-structured interview guide, conducted fortnightly in the first month post-discharge and monthly in the second and third months. Each session lasts 10–15 min, assessing exercise adherence and employing motivational interviewing techniques to identify barriers to exercise and propose solutions; **2. Provision of environmental cues**: Upon discharge, patients are given a leaflet containing a “Move Every Day, Healthy Heart” reminder card and simple exercise illustrations, to be displayed in a prominent location in their home to serve as behavioral prompts in daily life. **3. Long-term outcome assessment**: Instruct patients to complete the TSK-SV Heart, Barthel Index and SEE scales again during follow-up visits 1 month (T2) and 3 months (T3) after discharge to monitor the long-term maintenance of outcomes.

ADL, activities of daily living; CHD, coronary heart disease; NYHA, New York Heart Association; SEE, Self-Efficacy for Exercise scale; TSK-SV Heart, Tampa Scale for Kinesiophobia Heart.

### 2.3 Evaluation indicators

#### 2.3.1 Exercise anxiety

The Cardiac Patient Exercise Fear Scale was used for assessment. This scale was developed by Swedish researchers Bäck et al. (14) and adapted into Chinese by Lei et al. (13), comprising four dimensions and 17 items: perception of danger, avoidance of exercise, fear of exercise, and functional disruption. Each item is scored on a scale of 1–4, ranging from “strongly disagree” to “strongly agree”, with a total score of 17–68. A higher score indicates a greater level of exercise-related fear in the patient. Testing revealed that the Cronbach's α coefficient for this scale among CHD patients was 0.859, indicating good reliability and validity.

#### 2.3.2 Exercise self-efficacy

The Self-Efficacy for Exercise (SEE) scale was used for assessment. This scale was developed by Resnick et al. (15) and subsequently adapted into Chinese by Lee et al. (16). It primarily measures the patient's confidence in engaging in exercise when faced with obstacles. The scale comprises nine items, each scored on a scale of 0–10, ranging from “no confidence” to “very confident”. The total score is calculated as the mean of the nine item scores, with a total range of 0–10. A higher score indicates greater confidence in exercise. Testing revealed that the Cronbach's α coefficient for this scale among Chinese older adults was 0.75, making it suitable for the population in this study.

#### 2.3.3 Activities of daily living

Assessment was conducted using the Barthel Index (17), a commonly used measure of patients' activities of daily living (ADL). The scale comprises 10 items, with a maximum score of 100 indicating no dependence. 61–99 points indicate mild dependence, suggesting the patient can independently perform some daily activities; 41–60 points indicate moderate dependence, where the patient requires significant assistance to perform daily activities; and ≤ 40 points indicate severe dependence, where the patient requires full care from others for all daily activities. Testing revealed that the Cronbach's α coefficient for this scale in CHD patients was 0.93, demonstrating excellent reliability and validity.

#### 2.3.4 Safety

Adverse event criteria were established in accordance with the “Guidelines for the Diagnosis and Treatment of Coronary Heart Disease” (12), and the incidence of adverse events—including cardiovascular events (angina, arrhythmias, etc.,), exercise-related injuries, and falls—was recorded for both groups of patients during the intervention period.

#### 2.3.5 Exercise adherence

Exercise adherence was only assessed in the intervention group, defined as the proportion of days the home exercise programme was completed within 3 months of discharge. A rate of ≥80% was classified as high adherence, 50%−79% as moderate adherence, and <50% as low adherence. Exercise adherence was not measured in the control group, as they received only standard discharge advice without a structured home exercise prescription.

### 2.4 Data collection and quality control

Data collection will primarily rely on researchers who have undergone standardized training and members of the health belief team to guide patients in completing questionnaires and collect them on the spot, ensuring the accuracy of the information. Data will be collected on the day of admission (T0), the day of discharge (T1), 1 month post-discharge (T2), and 3 months post-discharge (T3). Prior to data collection, the study's content, objectives and methods were fully explained to participants. A one-to-one, face-to-face data collection method was adopted, strictly adhering to the principles of informed consent, beneficence and confidentiality. Following data collection, data were entered by two individuals and cross-checked in parallel to ensure accuracy.

### 2.5 Statistical methods

Data analysis was performed using SPSS 26.0 statistical software. Quantitative variables that followed a normal distribution were expressed as *x* ± s, and *independent t*-tests were used for intergroup comparisons; those that did not follow a normal distribution were expressed as *M* (*P*_25_, *P*_75_), and *Mann-Whitney U* tests were used for intergroup comparisons. Categorical variables were expressed as *n* (%), and chi-square tests or *Fisher's* exact test were used for intergroup comparisons. For repeated measures data, if the data follow a normal distribution and variances are homogeneous, repeated measures analysis of variance (ANOVA) was performed. The sphericity assumption was assessed using the Mauchly's test; if violated, Greenhouse-Geisser correction was applied. If there was no interaction between time and treatment factors, main effects were analyzed; if an interaction effect was present, separate effects were analyzed. Pairwise comparisons were corrected using the Bonferroni method (α' = 0.008). Missing data were handled according to the principle of the full data set.

## 3 Results

### 3.1 Comparison of baseline characteristics between the two groups

This study initially enrolled 140 patients. During the study, one patient in the experimental group was lost to follow-up, resulting in a final sample of 69 patients in the experimental group and 70 in the control group. Comparison of baseline characteristics between the two groups revealed no statistically significant differences (*P* > 0.05), indicating comparability. See Table 2 for details.

**Table 2:** Comparison of baseline characteristics between the two groups.

Variable	Group	Control group (*n* = 70)	Intervention group (*n* = 69)	*t/χ^2^/Fisher*	*P*
Age (years)		77.17 ± 7.18	76.07 ± 6.92	0.919	0.360
Gender				0.580	0.446
	Male	44	39		
	Female	26	30		
Marriage				-^*^	0.172
	Married/Living together	51	58		
	Unmarried	8	7		
	Widowed	10	3		
	Separated/Divorced	1	1		
Educational attainment				0.683	0.711
	Primary education or below	43	47		
	Lower secondary	17	14		
	Upper secondary/vocational school level	10	8		
Occupation				-^*^	0.174
	Farming	19	23		
	In employment	6	1		
	Retired	25	20		
	Unemployed	20	25		
Housing situation				0.372	0.830
	Living alone	10	9		
	Living with a partner	40	37		
	Living with children	20	23		
Income				-^*^	0.598
	Less than 3,000 yuan	48	49		
	3,001–5,000 yuan	17	18		
	Over 5,000 yuan	5	2		
Payment method				3.403	0.182
	Resident Medical Insurance	40	49		
	Employee health insurance	25	15		
	Other health insurance	5	5		
Smoking				2.129	0.145
	Yes	17	10		
	No	53	59		
Alcohol consumption				0.040	0.841
	Yes	11	10		
	No	59	59		
BMI				-^*^	0.964
	<18	6	5		
	18–24	52	50		
	24–28	8	9		
	>28	4	5		
Duration of CHD				0.210	0.900
	<1 year	32	34		
	1–3 years	16	14		
	>3 years	22	21		
Cardiac function class				3.758	0.153
	Class I	37	32		
	Grade II	23	18		
	Grade III	10	19		
Other chronic conditions				0.014	0.906
	None	24	23		
	Yes	46	46		

BMI, body mass index; CHD, coronary heart disease; NYHA, New York Heart Association. ^*^Fisher's exact test is used. This test has no test statistic, so it is denoted by “-” and reports exact P values.

### 3.2 Test for main effects of daily living abilities, fear of exercise and exercise self-efficacy between the two groups

**Figure 1:**
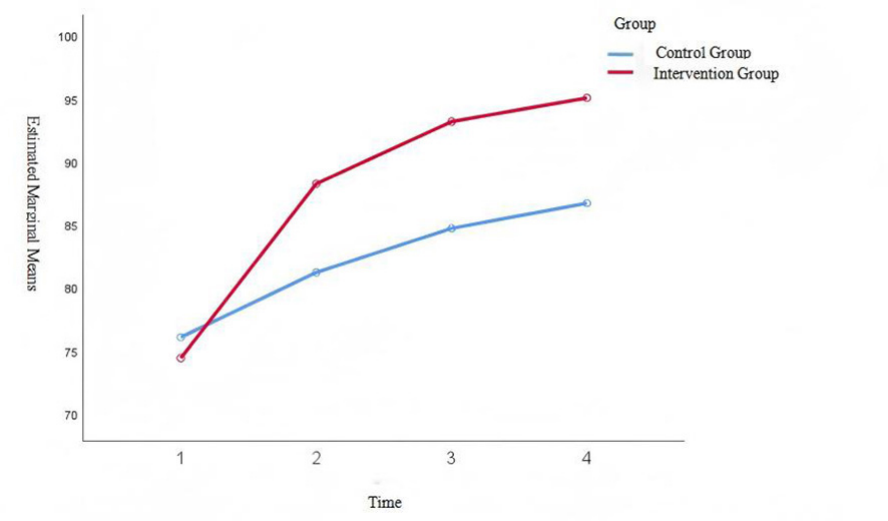
Interaction between the two patient groups and levels of activities of daily living at different time points.

**Figure 2:**
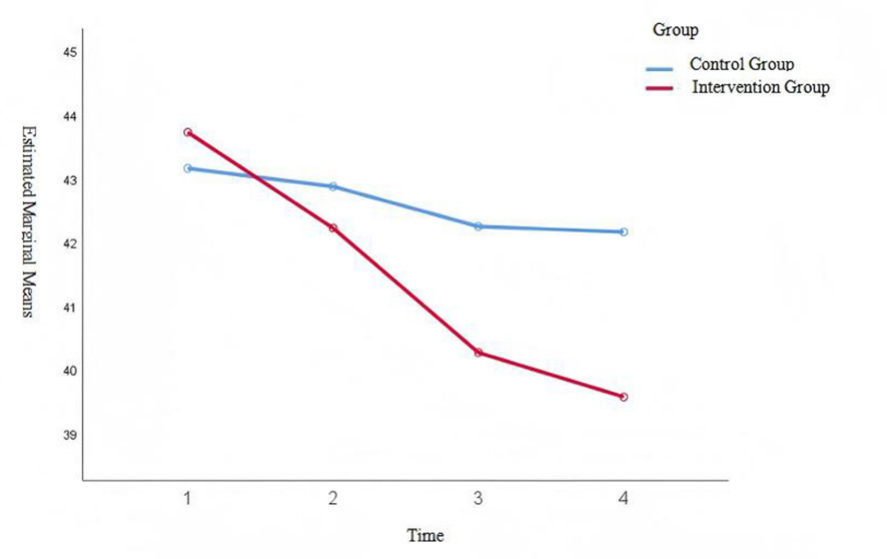
Interaction between exercise fear levels and group at different time points.

**Figure 3:**
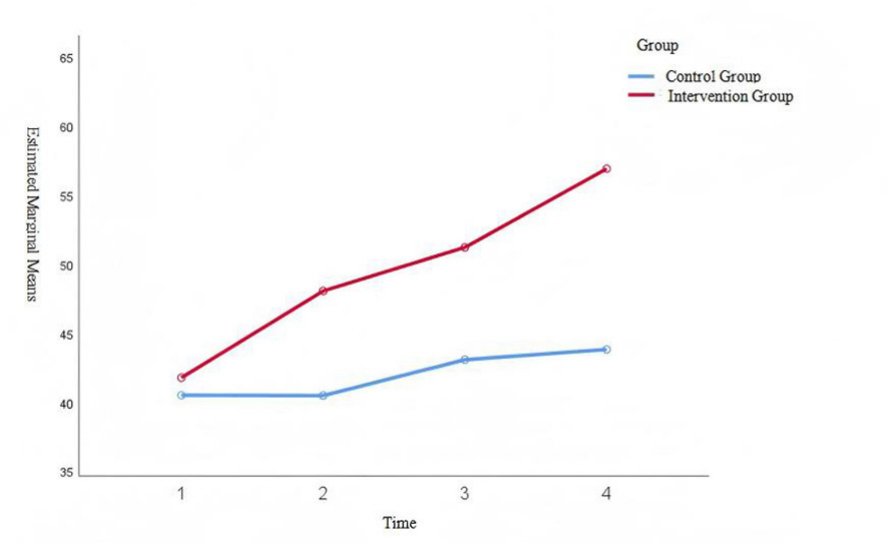
Interaction between exercise self-efficacy levels and group at different time points for the two patient groups.

Tests for normality and homogeneity of variance were conducted on the levels of activities of daily living, fear of exercise and exercise self-efficacy at various time points during the treatment period for the two groups of patients. The results showed that the levels of activities of daily living, fear of exercise and exercise self-efficacy in both groups followed a normal distribution and exhibited homogeneity of variance (*P* > 0.05). Mauchly's sphericity test indicated that the covariance matrices for ADL, exercise fear and exercise self-efficacy were not equal (χ^2^= 286.411, *P* < 0.001; χ^2^= 52.877, *P* < 0.001; χ^2^= 104.525, *P* < 0.001). Consequently, results were adjusted using the Greenhouse-Geisser method. Results of the repeated measures analysis of variance indicated that the time effects for ADL ability, fear of exercise and exercise self-efficacy were all significant (*P* < 0.05), meaning that the levels of these indicators changed over time; the between-group effects were significant (*P* < 0.05), indicating that there were differences in the levels of these indicators between the two groups; the interaction effects were significant (*P* < 0.05), suggesting that the influence of the time factor on these indicators varied depending on the treatment method; therefore, separate analyses of the time and between-group effects were conducted. See Table 3 and Figures 1–3 for details.

**Table 3:** Repeated measures analysis of variance for levels of activities of daily living, fear of exercise and exercise self-efficacy in the two groups of patients during treatment.

Measure	Time effect		Between-group effect		Interaction effect	
	*F*	*P*	*F*	*P*	*F*	*P*
Activities of daily living	75.475	<0.001	5.68	0.019	9.144	0.001
Fear of exercise	14.788	<0.001	9.399	0.003	5.218	0.003
Exercise self-efficacy	27.772	<0.001	6.518	0.012	10.488	<0.001

ADL, activities of daily living; *F, F*-statistic; *P, p*-value.

### 3.3 Tests of main effects of ADL ability, exercise fear and exercise self-efficacy in the two groups

Results of the time-specific effect tests indicate that, compared with pre-intervention levels, ADL ability in both groups increased following the intervention (*P* < 0.05). In the control group, there were no changes in exercise fear or exercise self-efficacy levels (*P* > 0.05), whereas in the experimental group, exercise fear levels decreased (*P* < 0.05) and exercise self-efficacy levels increased (*P* < 0.05). The levels of ADL in both the control and experimental groups increased with time (*P* < α'). In the experimental group, levels of exercise fear at 1 month and 3 months post-discharge were lower than on the day of admission and the day of discharge (*P* < α'). The experimental group's levels of exercise self-efficacy on the day of discharge, 1 month post-discharge, and 3 months post-discharge were all higher than on the day of admission (*P* < α'). See Table 4 for details.

**Table 4:** Comparison of activities of daily living, fear of exercise and self-efficacy for exercise between the two groups during treatment (x̄ ± s).

Measure	Group	Number of cases	On admission	On discharge	1 month after discharge	3 months after discharge	*F*	*P*
Activities of daily living	Control group	70	76.07 ± 18.71	81.21 ± 17.14a	84.71 ± 16.17ab	86.71 ± 16.39abc	9.831	<0.001
	Intervention group	69	74.42 ± 22.5	88.26 ± 14.45a	93.19 ± 9.35ab	95.07 ± 8.29abc	29.539	<0.001
	*F*		0.222	6.859	14.248	14.323		
	*P*		0.639	0.010	<0.001	<0.001		
Fear of exercise	Control group	70	43.16 ± 4.41	42.87 ± 3.88	42.24 ± 2.94	42.16 ± 3.33	0.884	0.451
	Intervention group	69	43.72 ± 4.3	42.22 ± 3.63	40.26 ± 3.53ab	39.57 ± 4.61ab	11.911	<0.001
	*F*		0.589	1.051	12.967	14.457		
	*P*		0.444	0.307	<0.001	<0.001		
Exercise self-efficacy	Control group	70	40.5 ± 17.01	40.47 ± 18.04	43.07 ± 21.56	43.81 ± 23.05	1.634	0.184
	Intervention group	69	41.77 ± 16.92	48.06 ± 17.76a	51.22 ± 18.29a	56.91 ± 18.26abc	21.103	<0.001
	*F*		0.194	6.242	5.764	13.766		
	*P*		0.660	0.014	0.018	<0.001		

ADL, activities of daily living; *F, F*-statistic; *P, p*-value; SD, standard deviation; SEE, self-efficacy for exercise scale.

Corrected using *the Bonferroni* method; the significance level for pairwise comparisons is α' = 0.008.

a indicates *P* < 0.008 compared with the day of admission; b indicates *P* < 0.008 compared with the day of discharge; c indicates *P* < 0.008 compared with 1 month after discharge.

Results of the group-specific effect tests showed that on the day of admission, there were no significant differences between the experimental and control groups in terms of ADL ability, exercise fear, or exercise self-efficacy (*P* > 0.05). The experimental group's ADL ability levels on the day of discharge, 1 month post-discharge, and 3 months post-discharge were all higher than those of the control group (*P* < 0.05). The exercise fear levels in the experimental group were lower than those in the control group 1 month and 3 months after discharge (*P* < 0.05), but there was no statistically significant difference between the experimental and control groups on the day of admission (*P* = 0.307). The exercise self-efficacy levels in the experimental group were higher than those in the control group on the day of admission, 1 month after discharge, and 3 months after discharge (*P* < 0.05). See Table 4 for details.

### 3.4 Safety in both groups and exercise adherence in the intervention group

During the intervention period, no exercise-related adverse events occurred in the intervention group. In the control group, two cases of angina pectoris were reported (both caused by patients increasing their exercise intensity on their own initiative), which resolved following symptomatic treatment. Among the 69 patients in the intervention group, 58 (84.06%) demonstrated high exercise adherence 3 months post-discharge, 9 (13.04%) demonstrated moderate adherence, and 2 (2.90%) demonstrated low adherence; overall adherence was good.

## 4 Discussion

### 4.1 The HBM-based cardiac rehabilitation intervention programme is scientifically sound, feasible, and has potential for wider application

The present study utilized the Health Belief Model (HBM) as its theoretical framework to construct a phased, structured cardiac rehabilitation programme for elderly CHD patients, following the sequence of “cognitive arousal → belief restructuring → behavioral practice → habit consolidation.” The present study systematically integrated the six core dimensions of the HBM into four intervention phases, thereby achieving a logical closed loop from cognitive intervention to behavioral shaping. This approach overcame the limitations of existing research, which lacked phased design and adaptability to the elderly population (18, 19).

The scientific rigor of the programme is attributable to its precise alignment with the mechanisms underlying kinesiophobia. Firstly, personalized risk education has been shown to address elderly patients' excessive perception of disease risk. Secondly, the integration of evidence-based lectures with supervised low-intensity exercise directly refutes the misconception that “exercise exacerbates the condition.” Thirdly, quantifiable short-term goals and specific feedback have been shown to accumulate successful experiences, targeting the low self-efficacy characteristic of elderly patients. All intervention measures are quantified and standardized (e.g., duration, pace, follow-up frequency), thereby enhancing reproducibility.

The results of the study confirmed the safety of the programme, as no exercise-related adverse events were observed, and its feasibility, with an overall adherence rate of 97.1%. Furthermore, the model does not necessitate the use of specialized equipment and can be implemented by nurses in primary care settings, thus providing a replicable model for hospitals with limited resources.

### 4.2 Comparison with previous studies

Our findings are consistent with earlier HBM-based interventions in chronic disease management. Komaç and Duru (10) showed that HBM education reduced cardiovascular risk factors in hypertensive patients, while Zhang et al. (11) reported improved glycaemic control in older adults with diabetes. However, unlike these single-domain programmes, our intervention integrated the HBM into a multi-phase cardiac rehabilitation protocol covering hospitalization and post-discharge follow-up. This comprehensive design may explain the sustained improvements in self-efficacy and activities of daily living (ADL).

Regarding kinesiophobia, Keessen et al. (5) and Çakal et al. (8) identified fear of movement as a major barrier to rehabilitation participation but did not test a targeted intervention. Our study provides evidence that a staged HBM approach can significantly reduce kinesiophobia, with a notable lag effect (no significant change at discharge but clear improvements at 1 and 3 months). This lag effect supports the fear-avoidance model (6), suggesting that cognitive restructuring requires time and repeated behavioral practice. Zhang et al. (18) also noted that single-phase in-hospital interventions may be insufficient for long-term change, reinforcing the value of our post-discharge follow-up component.

For exercise self-efficacy, our results align with Bandura's theory, which posits that mastery experiences, vicarious experience, verbal persuasion, and emotional arousal are key sources of self-efficacy (20–22). The gradual increase in walking duration (“5 min more each day”) helped patients accumulate mastery experiences, while group sessions provided vicarious experience. This multi-pathway approach likely contributed to the sustained increase in self-efficacy observed in the intervention group.

The improvement in ADL is clinically meaningful. Hou et al. (23) and Yifan et al. (24) reported that kinesiophobia independently predicts ADL decline in older cardiac patients. Our findings extend this evidence by showing that directly targeting kinesiophobia can reverse that decline, with the intervention group achieving higher ADL scores at discharge and sustained improvement over 3 months.

### 4.3 Effects on kinesiophobia and exercise self-efficacy

The intervention group showed a statistically significant reduction in kinesiophobia at 1 and 3 months post-discharge, whereas the control group exhibited no change. This finding aligns with previous studies that identified kinesiophobia as a major barrier to rehabilitation participation (5, 8). The Borg RPE-guided exercise prescription (target 11–13, “fairly light to somewhat hard”) appeared safe and well tolerated, with no exercise-related adverse events. The observed lag effect—improvements only after discharge—highlights the importance of continued follow-up. We hypothesize that during hospitalization, patients may still rely on medical supervision, whereas post-discharge, they must independently apply learned behaviors, leading to a consolidation of benefits. This interpretation is consistent with Zhang et al. (18), who noted that single-phase in-hospital interventions are often insufficient for long-term behavioral change.

For exercise self-efficacy, the intervention group demonstrated a progressive increase from discharge to 3 months, while the control group showed no significant change. This sustained upward trend can be explained by Bandura's theory, which identifies mastery experiences, vicarious experience, verbal persuasion, and emotional arousal as key sources of self-efficacy (20–22). The systematic use of specific feedback (“You completed a 10-min walk under supervision; your heart rate and blood pressure remained stable”) provided mastery experiences, while group sessions offered vicarious experience. Together, these pathways likely contributed to the long-term improvement in self-efficacy observed in the intervention group.

### 4.4 Effects on activities of daily living

The improvement in ADL in the intervention group (from 74.4 at admission to 95.1 at 3 months) was significantly larger than in the control group (from 76.1 to 86.7). Previous research has shown that kinesiophobia independently predicts ADL decline in older cardiac patients (23, 24). Our findings extend this evidence by demonstrating that directly targeting kinesiophobia can reverse that decline. The greater improvement in ADL likely reflects both reduced fear of movement and enhanced physical capacity through walking and supplementary exercises. Importantly, the gap between groups widened over time, suggesting that the HBM-based intervention successfully transformed hospital-acquired exercise capacity into stable daily living behaviors. This finding supports the notion that ADL improvement should not be viewed solely as a rehabilitation endpoint but as a core objective of continuity of care after discharge (23).

### 4.5 Research features and limitations

The innovation of this study is threefold: (1) personalized, phased intervention design operationalizing the HBM across four sequential stages; (2) rigorous repeated-measures RCT design; and (3) a practical, nurse-led model that integrates in-hospital initiation with home-based continuation.

Several limitations should be acknowledged. First, the single-center design and limited sample size may restrict generalizability. Second, the follow-up period was only 3 months; longer-term effects (≥12 months) remain unknown. Third, objective functional capacity measures (e.g., six-minute walk test, peak oxygen uptake) were not included; our study focused on psychological and ADL outcomes. Fourth, we did not systematically document the completeness of coronary revascularization. Residual small-vessel disease or microvascular angina might still contribute to kinesiophobia, even when large-vessel revascularization is considered complete. Future multicenter trials with extended follow-up, objective functional testing, and detailed revascularization data are warranted.

Despite these limitations, the programme is safe, feasible, and ready for implementation in resource-limited primary care settings. It provides evidence-based support for incorporating psychological strategies into standard cardiac rehabilitation pathways for older adults.

## 5 Conclusion

The present study was a single-center trial with a limited sample size and a relatively short follow-up period. These factors had a somewhat detrimental effect on the generalizability of the results and the validation of long-term benefits. It is recommended that future research be deepened in the following directions: Firstly, multicenter, large-sample, randomized controlled trials should be conducted, with the follow-up period extended to 6 months or 1 year, and multidimensional outcome measures incorporated to comprehensively assess the long-term benefits of the intervention. Secondly, statistical methods such as structural equation modeling should be utilized to validate the mediating pathway of “fear of exercise—self-efficacy—ADL,” thereby further elucidating the intrinsic mechanisms of action between the various dimensions of the Health Belief Model. Thirdly, explore remote rehabilitation models integrating “Internet+” with the HBM should be explored, and intelligent intervention platforms based on mobile applications developed to meet the ongoing rehabilitation needs of discharged patients more efficiently and conveniently. Fourthly, qualitative research should be conducted to delve deeper into patients' subjective experiences and underlying motivations regarding shifts in their beliefs, thereby providing richer evidence-based grounds for optimizing intervention models. This study integrates the Health Belief Model into cardiac rehabilitation for elderly CHD patients. It validates the efficacy and safety of a staged, HBM-based intervention programme. The findings suggest that the programme is efficacious in reducing exercise fear levels in elderly patients with coronary heart disease, whilst concurrently enhancing their exercise self-efficacy and activities of daily living (ADL) capabilities. Furthermore, as the intervention measures are quantifiable and standardized, they possess strong clinical feasibility and potential for widespread implementation. This study provides evidence-based support for the development of psychologically oriented cardiac rehabilitation strategies for elderly patients with CHD. It is recommended that this programme be incorporated into the standard service protocols for cardiac rehabilitation of elderly CHD patients in clinical practice, thereby promoting multidisciplinary collaboration and nurse-led cardiac rehabilitation models. Concurrently, the outpatient continuity of care system should be enhanced to provide comprehensive, personalized rehabilitation support for elderly patients.

## Data Availability

The original contributions presented in the study are included in the article/supplementary material, further inquiries can be directed to the corresponding author.
